# Dietary mineral supplies in Africa

**DOI:** 10.1111/ppl.12144

**Published:** 2014-02-13

**Authors:** Edward J M Joy, E Louise Ander, Scott D Young, Colin R Black, Michael J Watts, Allan D C Chilimba, Benson Chilima, Edwin W P Siyame, Alexander A Kalimbira, Rachel Hurst, Susan J Fairweather-Tait, Alexander J Stein, Rosalind S Gibson, Philip J White, Martin R Broadley

**Affiliations:** a School of Biosciences, University of Nottingham, Sutton Bonington CampusLoughborough LE12 5RD, UK; b British Geological SurveyKeyworth, Nottingham NG12 5GG, UK; c Ministry of Agriculture and Food SecurityLunyangwa Research Station, P.O. Box 59, Mzuzu, Malawi; d Community Health Sciences Unit, Ministry of HealthPrivate Bag 65, Lilongwe, Malawi; e Department of Human Nutrition and Health, Lilongwe University of Agriculture and Natural ResourcesP.O. Box 219, Lilongwe, Malawi; f Norwich Medical School, University of East AngliaNorwich NR4 7TJ, UK; g IFPRI2033 K Street NW, Washington, DC 20006, USA; h Department of Human Nutrition, University of OtagoP.O. Box 56, Dunedin, New Zealand; i Ecological Sciences, The James Hutton InstituteInvergowrie, Dundee DD2 5DA, UK

## Abstract

Dietary micronutrient deficiencies (MNDs) are widespread, yet their prevalence can be difficult to assess. Here, we estimate MND risks due to inadequate intakes for seven minerals in Africa using food supply and composition data, and consider the potential of food-based and agricultural interventions. Food Balance Sheets (FBSs) for 46 countries were integrated with food composition data to estimate per capita supply of calcium (Ca), copper (Cu), iron (Fe), iodine (I), magnesium (Mg), selenium (Se) and zinc (Zn), and also phytate. Deficiency risks were quantified using an estimated average requirement (EAR) ‘cut-point’ approach. Deficiency risks are highest for Ca (54% of the population), followed by Zn (40%), Se (28%) and I (19%, after accounting for iodized salt consumption). The risk of Cu (1%) and Mg (<1%) deficiency are low. Deficiency risks are generally lower in the north and west of Africa. Multiple MND risks are high in many countries. The population-weighted mean phytate supply is 2770 mg capita^−1^ day^−1^. Deficiency risks for Fe are lower than expected (5%). However, ‘cut-point’ approaches for Fe are sensitive to assumptions regarding requirements; e.g. estimates of Fe deficiency risks are 43% under very low bioavailability scenarios consistent with high-phytate, low-animal protein diets. Fertilization and breeding strategies could greatly reduce certain MNDs. For example, meeting harvestplus breeding targets for Zn would reduce dietary Zn deficiency risk by 90% based on supply data. Dietary diversification or direct fortification is likely to be needed to address Ca deficiency risks.

## Introduction

Dietary mineral micronutrient deficiencies (MNDs), including calcium (Ca), copper (Cu), iron (Fe), iodine (I), magnesium (Mg), selenium (Se) and zinc (Zn) are likely to be widespread in global terms (Black et al. 2008, Broadley and White 2010, Bouis et al. 2011, Muthayya et al. 2013). Determining the prevalence of these and other MNDs (collectively termed *hidden hunger*) is an immense challenge. For some micronutrients, such as Fe, Se and Zn, mineral status can be analyzed directly via whole blood or plasma fractions or indirectly via activities of micronutrient-responsive enzymes (Gibson 2005, Fairweather-Tait et al. 2011). However, collection of blood is invasive and time-consuming, with reliable and expensive analytical equipment required for elements such as Se, whose detection limits are in the low parts-per-billion range. By contrast, other micronutrients such as I are monitored reliably in other tissues or in urine (Zimmermann 2008). MND risks due to inadequate intake can also be quantified by direct intake assessments from duplicate diets (Hurst et al. 2013) or from dietary surveys (Gibson and Huddle 1998, Department of Health/Food Standards Agency 2011, Ecker and Qaim 2011). Unfortunately, many countries do not have data available from nationally representative dietary analyses or surveys. Furthermore, data from surveys can be affected by behavioral change and misreporting (Vuckovic et al. 2000, Rennie et al. 2007, Ovaskainen et al. 2008, Archer et al. 2013).

An alternative method for estimating MND risks at wide scales is to use per capita food supplies from food balance sheets (FBSs) as a proxy for food consumption (de Haen et al. 2011). These data can be combined with food composition tables, with the resulting values of micronutrient supply being compared to an estimated average requirement (EAR) ‘cut-point’ for specific micronutrients and populations. This indirect method is similar to that used by the United Nations Food and Agriculture Organization (FAO) to estimate the global prevalence of undernutrition (FAO 2012a) and has been adopted previously to estimate the global prevalence of Zn deficiencies (Wuehler et al. 2005, Wessells and Brown 2012) and the prevalence of Mg and Se deficiency in Africa (Hurst et al. 2013, Joy et al. 2013). FBSs are currently available for all years between 1961 and 2009 (FAO 2012b) and are based on national production statistics, adjusted for imports/exports, losses during transport, storage and processing, non-food uses such as industrial products and livestock feed and food stock balances (FAO 2001). These data are presented by the FAO on a mean per capita basis in terms of product weight (as consumed or traded) and assume that the net available food supply is consumed in full with no adjustment for household waste. To determine per capita micronutrient supply, FBSs can be multiplied by their micronutrient concentration as reported in food composition tables and compared to an EAR ‘cut-point’. The EAR of each micronutrient is defined as the daily nutrient intake estimated to meet the needs of half the healthy individuals in a specific sex and life-stage group (World Health Organization, WHO/FAO 2004). In previous studies, inter- and intra-household economic, social or gender inequalities were assumed to be captured by a 25% inter-individual coefficient of variation (cv), which is distributed normally around the estimated national mean per capita supply.

Previous studies, based on food supply data for 2007 and regionally assembled food composition data from several sources, have shown that the risk of Mg deficiency seems to be low (<4%) in Africa (Joy et al. 2013). The risk of Se deficiency was much greater, at 22% for Africa as a whole and >60% in eight countries (Hurst et al. 2013). Assessing the risk of Se deficiency is challenging due to limited food composition data for Se and likely effects of spatial variation in soil geochemistry (Chilimba et al. 2011).

Supply-based approaches have not been adopted widely for most MNDs. This study aimed to extend previous analyses to estimate the risk of Ca, Cu, Fe, I, Mg, Se and Zn deficiency in Africa based on food supply data for 2009. We also sought to determine the dietary supply of phytate. Typically, 60–80% of the phosphorus (P) content of seeds occurs as mixed salts of phytic acid (myo-inositol hexakisphosphate, IP_6_; Raboy, 2009), collectively termed as phytate, which humans and monogastric animals such as pigs and poultry cannot digest (Brinch-Pedersen et al. 2002). Given that phytate inhibits Ca, Fe, Mg and Zn absorption in the human intestine (Miller et al. 2007, Hurrell and Egli 2010) and that crop breeding can reduce the phytate content of cereals and legumes (Raboy 2002), it may be appropriate to consider lower phytate crops to complement other plant nutritional management strategies such as biofortification through micronutrient fertilization or breeding (White and Broadley 2009, Bouis and Welch 2010). However, as dietary phytate intake has been linked to health benefits including reduced risks of cancer and osteoporosis (Vucenik and Shamsuddin 2006, López-González et al. 2008), reductions in phytate in human diets should proceed with caution.

## Materials and methods

Per capita supply of Ca, Cu, Fe, I, Mg, Se and Zn, phytate and macronutrients, such as fat, protein and total available carbohydrate, was estimated for 46 countries in Africa, as the product of food supply and food composition data. Deficiency risks were estimated using an EAR ‘cut-point’ for each element.

### Food supply data

Food supply data were sourced from FAO FBSs for 2009 (FAO 2012b). FBS datasets provide estimates of the annual food supply at a retail level for up to 92 separate food items, e.g. ‘Maize’, ‘Tomatoes’, ‘Freshwater Fish’, ‘Bovine Meat’ etc. Most countries in Africa were included, except for those lacking FBS data (Equatorial Guinea, Somalia, Western Sahara, Mayotte, Réunion and Saint Helena) or small island states (Cape Verde, Comoros, Mauritius, Sao Tome and Principe and Seychelles) because of their likely high dependence on imports and exports. Thus, 2009 data were sourced for 45 countries. For Democratic Republic of Congo (DRC), 2007 FBS data were used (FAO 2011) as 2009 data were unavailable.

### Food composition data

**Figure 1:**
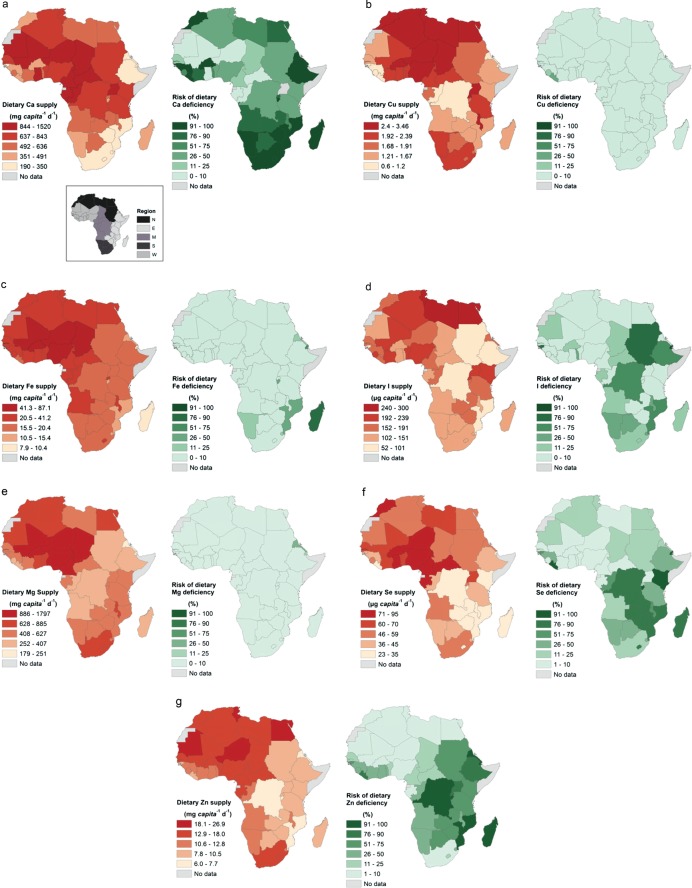
Micronutrient supply and risk of deficiency due to inadequate intake for 46 African countries. Inset: regions are based on UN sub-regions (http://unstats.un.org/unsd/methods/m49/m49regin.htm)

Data were sourced for energy, micronutrients, macronutrients and phytate from published food composition tables. As described previously (Joy et al. 2013), the initial aim was to create five regional databases, covering all foods in the FBSs, which mapped onto the five United Nations (UN) African sub-regions: ‘Northern’ (N), ‘Eastern’ (E), ‘Southern’ (S), ‘Middle’ (M) and ‘Western’ (W) Africa (Fig. 1, Table S1). However, because published large-scale food composition tables were not identified for Northern and Middle Africa, three databases were generated: Eastern, Southern (also used for Northern) and Western (also used for Middle) Africa. The Southern database was used for Northern Africa because both regions are in the sub-tropics and share many biophysical features. The Western database was used for Middle Africa as both are situated primarily within the tropics. Food composition tables containing the greatest number of food items were preferred. Tables were searched to find a ‘best-fit’ match with FBS items based on the judgment of the authors. A single data point was selected for each item in each regional food composition table with no averaging of data from different sources so that data points can be traced back to the original source and fitted item. Data for Eastern Africa were sourced primarily from ‘Tanzanian’ food composition tables (Lukmanji et al. 2008) complemented by data from Malawi (Ferguson et al. 1989, Donovan et al. 1991) and Mozambique (Korkalo et al. 2011). The Western Africa data were sourced primarily from Stadlmayr et al. (2010), complemented by data from Mali (Barikmo et al. 2004), Gambia (Prynne and Paul 2011) and Ghana (Ferguson et al. 1993). The Southern Africa data were sourced primarily from Wolmarans et al. (2010); other sources contributed small amounts of composition data (Eckhoff and Maage 1997, Abebe et al. 2007, Mbata et al. 2009). Where suitable food concentration data could not be identified, data from the other regions were used. Any remaining data gaps were filled using US (USDA-ARS 2011) or UK (FSA 2002) food composition data. An important caveat is that some of the food composition data are based on secondary sources. For example, Lukmanji et al. (2008) include data derived from multiple sources, including US food composition data, whereas the Malawi and Ghana sources reflect primary analysis of local foods. Detailed consideration of primary and secondary sources of data is outside the scope of this study given the difficulty of tracking items back to their original source. There are also some data quality issues in the food composition tables. For example, in a previous study (Hurst et al. 2013), we used Se composition data for the FBS item ‘Coconuts – Incl Copra’ from Stadlmayr et al. (2010). As discussed in that paper, the value of 810 µg (100 g)^−1^ is likely to be unrealistically high, suggesting either measurement or reporting error. Therefore, in this study, we use the US food composition value of 10.1 µg (100 g)^−1^ (USDA-ARS 2011). All concentrations are expressed per 100 g edible portion, either on a dry-weight or fresh-weight basis depending on how supply of FBS items is measured. Regional allocation of country, food concentration data, literature sources and best-fit FBS categories are shown in Tables S1 and S2. These tables can be audited and updated with improved (e.g. spatially disaggregated) food composition data in the future.

### Adjusting food iodine supply estimates based on iodized salt usage

The I concentration of salt was estimated at a national scale based on the proportion of households consuming iodized salt and its level of iodization (UNICEF 2012). Per capita salt consumption was estimated from regional salt supply data from the International Council for the Control of Iodine Deficiency Disorders (ICCIDD 2006) (Table S3). Fortification schemes for other minerals (e.g. Fe) were not considered due to lack of data on coverage and compliance.

### Demographic data

Demographic data were sourced from the UN which provides national estimates of population by sex, age distribution and number of births within each 5-year age group for 2010 (UN 2011). The crude pregnancy rate was calculated for each age group as the crude birth rate (CBR) × 280/365. Lactation was assumed to last for 2 years (i.e. CBR × 2), although this is likely to be an overestimate due to infant mortality and cessation of breastfeeding prior to 24 months.

### Dietary requirements

To estimate dietary requirements, a national EAR was set for each micronutrient (Ca, Cu, Fe, I, Mg, Se and Zn) using WHO and Institute of Medicine (IOM) data (IOM 2000, 2002, WHO/FAO 2004). A mean EAR of the national population was calculated and weighted according to country-specific demographic data (UN 2011), because EARs are specific to sex and life-stage group (Tables S4 and S5). An EAR can be derived from the reference nutrient intake (RNI), the intake level of a specific micronutrient that is sufficient for approximately 97% of a specific sex and life-stage group. RNIs provided in WHO/FAO (2004) were converted to EARs using standard conversion factors (WHO/FAO 2006, p. 292). Preference was given to WHO requirement data as these are applicable to non-US population groups. Where WHO data were lacking (i.e. for carbohydrate, fat, fiber and Cu requirements), US dietary reference intake values were used. The US/IOM values were also used for Se (e.g. adult EAR = 45 µg) because WHO recommendations for Se intake (e.g. adult male EAR = 28 µg day^−1^; WHO/FAO 2004) are probably too low based on recent evidence (Fairweather-Tait et al. 2011); however, use of WHO values is explored in the Discussion Section. The EAR for Ca was set to account for diets with low-animal protein intake relative to Western intake levels (e.g. adult male EAR = 625 mg day^−1^; WHO/FAO 2004). The EARs for Fe (adult female EAR = 13.4 mg day^−1^) and Zn (adult female EAR = 8.2 mg day^−1^) were based on ‘low dietary bioavailability’ which is appropriate for most diets in developing countries and assumes 10% bioavailability for Fe and 15% for Zn (WHO/FAO 2004). Use of very low dietary bioavailability for Fe (5%) is explored in the Discussion Section. A total net Fe requirement of 840 mg during pregnancy was used to estimate the additional daily Fe requirement (WHO/FAO 2004, p. 264). Pregnancy was assumed to last for 280 days, with an average 50% increase in Fe bioavailability from 10 to 15% due to homeostatic responses. Pregnant women were therefore assumed to require 20 mg Fe day^−1^ in addition to their normal requirements. The Zn EAR during pregnancy was estimated to be the average requirement for the first, second and third trimesters (i.e. 12.5 mg day^−1^); during lactation, the EAR for Zn was the weighted average of 0–3, 3–6 and 6+ months, assuming 24 months of lactation (i.e. 12.8 mg day^−1^). For pregnant and lactating women aged 15–19 years, the Zn EARs during both the third trimester and 0–3 months of lactation periods were assumed to be double to those of non-pregnant women of the same age group (WHO/FAO 2004, p. 238). Women were assumed to have no increased Mg requirement during pregnancy, but a 42 mg day^−1^ increase in EAR was observed when lactating.

### Data integration

The risks of MNDs were estimated using an EAR ‘cut-point’, and those at risk are assumed to be the proportion of the population with intakes below the EAR (Carriquiry 1999). The FBS and food composition data were integrated using standard database queries (microsoft access 2010, Microsoft Corporation, Redmond, WA). A per capita supply of each micronutrient was estimated for each country in Africa based on the product of food supply and composition (Table S6). Daily intake within populations was assumed to follow a normal distribution based on mean per capita supply and a cv of 25% to account for inter-individual variation in intake, as in a previous study (Joy et al. 2013). Iodine supply was estimated as the sum of supply from food and salt sources. Data were mapped using arcgis (Version 9.3, ESRI, Redlands, CA). Subsequently, FBS items were assigned to six high-level food groups (‘Animal Products’, ‘Cereals’, ‘Fruits and Vegetables’, ‘Pulses and Beans’, ‘Roots and Tubers’, and ‘Other foods’; Table S8) so that the contribution of each food group to micronutrient supplies could be assessed.

## Results

Micronutrient supply and estimated MND risks due to inadequate intake for 46 African countries are mapped in Fig. 1; data are provided in detail in Tables S6 and S7, along with macronutrient and phytate supply. Micronutrient supply data and MND risks are summarized by region in Table1.

**Table 1:** Estimated supply and deficiency, by region and overall, for 46 African countries. EAR, estimated average requirement; M, million

Region	2010 population	Energy supply	Calcium				Copper				Iron			
			EAR	Supply	Deficiency		EAR	Supply	Deficiency		EAR	Supply	Deficiency	
	M	(kcal capita^−1^ day^−1^)	mg capita^−1^ day^−1^		%	M	mg capita^−1^ day^−1^		%	M	mg capita^−1^ day^−1^		%	M
Northern	208.9	3510	643	599	62	129.1	0.61	2.59	0.1	0.3	10.7	22.8	2.1	4.3
Eastern	311.5	2390	636	583	69	215.0	0.58	1.80	0.6	1.9	10.3	16.8	14.1	43.9
Southern	57.8	3360	645	353	99	57.3	0.61	1.93	0.3	0.2	10.7	18.8	4.6	2.7
Western	303.8	3261	633	746	36	108.9	0.58	2.39	1.1	3.5	10.3	44.3	0.3	0.9
Middle	125.8	2355	632	764	31	38.6	0.58	1.44	3.7	4.7	10.2	26.2	1.6	2.0
Mean (population-weighted)		2937	636	645	54		0.59	2.11	1.1		10.4	27.6	5.3	
National median of 46 countries		2723	635	574	67		0.59	1.84	0.3		10.4	22.8	1.6	
Total	1007.8					549.0				10.6				53.8

**Table:** 

Region	Iodine				Magnesium				Selenium				Zinc			
	EAR	Supply	Deficiency		EAR	Supply	Deficiency		EAR	Supply	Deficiency		EAR	Supply	Deficiency	
	µg capita^−1^ day^−1^		%	M	mg capita^−1^ day^−1^		%	M	µg capita^−1^ day^−1^		%	M	mg capita^−1^ day^−1^		%	M
Northern	102	205	18.2	39.2	169	607	0.5	1.1	39	58	12.2	25.5	10.4	16.2	16	33.2
Eastern	101	147	26.3	81.8	158	478	1.2	3.6	37	39	52.3	163.0	10.5	8.9	75	233.8
Southern	103	123	25.5	14.8	170	621	0.2	0.1	39	48	26.1	15.1	10.4	15.9	10	6.1
Western	101	201	4.6	14.0	158	994	0.1	0.4	37	75	5.5	16.8	10.5	15.4	17	50.8
Middle	101	127	32.5	40.9	156	552	1.2	1.5	37	43	49.4	62.1	10.5	9.6	64	80.5
Mean (population-weighted)	101	171	18.9		161	678	0.7		38	55	28.0		10.5	12.9	40	
National median of 46 countries	101	157	8		160	569	0.2		38	50	17.2		10.5	11.5	36	
Total				190.7				6.8				282.4				404.2

### Energy supply

**Figure 2:**
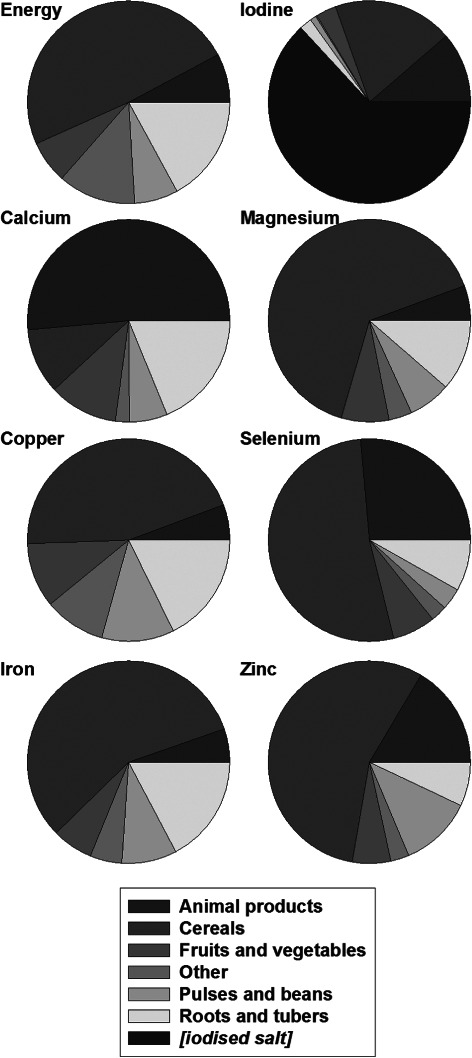
Proportional contribution of the major food groups to dietary energy and micronutrient supply for 46 African countries.

From a total population of 1.01 billion, mean energy supply from all food sources (population-weighted) was 2937 kcal capita^−1^ day^−1^, ranging at a national scale from 1679 (Eritrea) to 3851 (Egypt) kcal capita^−1^ day^−1^ (Table S7). Energy supply was considerably lower in the Middle and Eastern regions than in the Northern, Western and Southern regions (Table1). ‘Cereals’ and ‘Roots and Tubers’ contributed two-thirds of energy supply, while ‘Animal Products’ contributed 8% (Fig. 2, Tables S8–S10). The contribution of ‘Cereals’ to total energy supply ranged from 16 (Central African Republic) to 81% (Lesotho); ‘Roots and Tubers’ ranged from 1 (Gambia) to 61% (DRC) and ‘Animal Products’ from 3 (DRC) to 19% (Mauritania).

### Calcium

The estimated mean risk of dietary Ca deficiency throughout Africa was 54%, the greatest deficiency risk of all micronutrients studied (Tables1 and S6) and was >95% in 16 of the 46 countries examined. The risk of Ca deficiency was greatest in the Southern region (99%), followed by Eastern (69%), Northern (62%), Western (36%) and Middle (31%) regions. Compared to a mean EAR of 636 mg capita^−1^ day^−1^, the mean Ca supply (population-weighted) was 645 mg capita^−1^ day^−1^, ranging from 190 (Lesotho) to 1518 mg capita^−1^ day^−1^ (Uganda). The median national Ca supply was 574 mg capita^−1^ day^−1^. Among the higher level food groups, ‘Animal Products’ provided the greatest single contribution to Ca supply (approximately 50%; Fig. 2, Tables S8–S10), ranging from 19 (Côte d’Ivoire) to 88% (Mauritania). Further disaggregation showed dairy products to be especially important sources of Ca in the Northern and Southern regions, supplying >50% of Ca, while fish supplied 40, 38 and 30% of Ca in the Eastern, Middle and Western regions, respectively. ‘Roots and Tubers’ (notably cassava) were important sources of Ca in the Western and Middle regions, e.g. supplying 51% of Ca in Côte d’Ivoire. ‘Fruits and Vegetables’ were important sources of Ca, particularly in Northern Africa, e.g. supplying 39% of Ca in Egypt and 36% in Morocco. National per capita energy and Ca supply were not significantly correlated, (R^2^ = 0.04, *P* = 0.10). If all of Africa was to meet its RNI for Ca, this would require 769 t day^−1^ of mineral Ca, approximately 130 t more than the current supply. This is the equivalent of 60–120 million L day^−1^ of milk depending on its Ca concentration.

### Copper

The mean estimated risk of Cu deficiency was 1.1% (Tables1 and S6). Compared to a mean EAR of 0.59 mg capita^−1^ day^−1^, mean Cu supply (population-weighted) was 2.11 mg capita^−1^ day^−1^, ranging from 0.60 (Liberia) to 3.46 mg capita^−1^ day^−1^ (Niger). The median national Cu supply was 1.84 mg capita^−1^ day^−1^. On average, ‘Cereals’ contributed 45% of Cu supply throughout Africa (Fig. 2, Tables S8–S10), ranging from 8 (Rwanda) to 85% (Burkina Faso). National per capita energy and Cu supply were positively correlated (R^2^ = 0.34, *P* < 0.001).

### Iron

The mean estimated risk of Fe deficiency was 5% and was greatest in the Eastern region (14%) followed by the Southern (5%), Northern (2%), Middle (2%) and Western (<1%) regions (Tables1 and S6). Compared to a mean EAR of 10.4 mg capita^−1^ day^−1^, mean Fe supply (population-weighted) was 27.6 mg capita^−1^ day^−1^, ranging from 7.9 (Madagascar) to 87.1 mg capita^−1^ day^−1^ (Niger). The median national Fe supply was 22.8 mg capita^−1^ day^−1^, of which ‘Cereals’ provided 57% (Fig. 2, Tables S8–S10), ranging from 12 (Rwanda) to 89% (Eritrea). ‘Animal Products’ generally made a small contribution to total Fe supply (5%), although this was 14% in the Southern region. National per capita energy and Fe supply were positively correlated (R^2^ = 0.12, *P* = 0.012).

### Iodine

The mean estimated risk of I deficiency (after accounting for iodized salt consumption) was 19% and was greatest in the Middle region (33%), followed by the Eastern (26%), Southern (26%), Northern (19%) and Western (5%) regions (Tables1 and S6). Compared to a mean EAR for I of 101 µg capita^−1^ day^−1^, the mean I supply (population-weighted) was 171 µg capita^−1^ day^−1^, ranging from 52 (Djibouti) to 300 µg capita^−1^ day^−1^ (Tunisia). The median national I supply was 157 µg capita^−1^ day^−1^. The supply of I was dominated by iodized salt, which contributed 63% of the total supply in Africa. Estimates of the use of sufficiently iodized salt (>15 ppm) ranged from 0% of households (Djibouti) to 98% (Kenya). Without I supply through salt, 89% of the population of Africa would be at risk of I deficiency due to inadequate dietary supply. The contribution of food to total I supply is important as ‘Cereals’ and ‘Animal Products’ contributed 19 and 11%, respectively, of the total I supply in Africa (Tables S8–S10). This high contribution of cereals to I intake is perhaps surprising given that this element is not readily translocated to cereal grains; there is need for further grain I composition data to confirm this finding. National per capita energy and I supply (from food and salt) were positively correlated (R^2^ = 0.20, *P* = 0.001).

### Magnesium

The mean estimated risk of Mg deficiency was 0.7% (Tables1 and S6). Compared to a mean EAR for Mg of 161 mg capita^−1^ day^−1^, the mean Mg supply (population-weighted) was 678 mg capita^−1^ day^−1^, ranging from 179 (Eritrea) to 1797 mg capita^−1^ day^−1^ (Burkina Faso); the median national Mg supply was 569 mg capita^−1^ day^−1^. ‘Cereals’ and ‘Roots and Tubers’ contributed 65 and 11%, respectively, of the Mg supply across the continent (Fig. 2, Tables S8–S10). National per capita energy and Mg supply were positively correlated (R^2^ = 0.15, *P* = 0.004).

### Selenium

The mean estimated risk of Se deficiency was 28% and was greatest in the Eastern region (52%), followed by the Middle (49%), Southern (26%), Northern (12%) and Western (6%) regions (Tables1 and S6). Compared to a mean EAR for Se of 38 µg capita^−1^ day^−1^, the mean Se supply (population-weighted) for Africa was 55 µg capita^−1^ day^−1^, ranging from 23 (Liberia) to 93 µg capita^−1^ day^−1^ (Burkina Faso). The median national Se supply was 50 µg capita^−1^ day^−1^. ‘Cereals’ contributed 52% of Se supply throughout Africa (Fig. 2, Tables S8–S10). ‘Animal Products’ were an important source of Se in the Northern (34%) and Southern (62%) regions. National per capita energy and Se supply were positively correlated (R^2^ = 0.32, *P* < 0.001).

### Zinc

The mean estimated risk of Zn deficiency was 40% (Tables1 and S6) and was greatest in the Eastern region (75%), followed by the Middle (64%), Western (17%), Northern (16%) and Southern (10%) regions. Compared to a mean EAR for Zn of 10.5 mg capita^−1^ day^−1^, the mean Zn supply (population-weighted) was 12.9 mg capita^−1^ day^−1^, ranging from 6.0 (Eritrea) to 26.9 mg capita^−1^ day^−1^ (Niger). The median national Zn supply was 11.5 mg capita^−1^ day^−1^. ‘Cereals’ contributed 56% of Zn supply across the continent (Fig. 2, Tables S8–S10), ranging from 18 (Rwanda) to 82% (Lesotho), while ‘Animal Products’ (16%) and ‘Pulses and Beans’ (12%) are also important sources of Zn supply. National per capita energy and Zn supply were strongly positively correlated (R^2^ = 0.50, *P* < 0.001).

### Phytate

**Figure 3:**
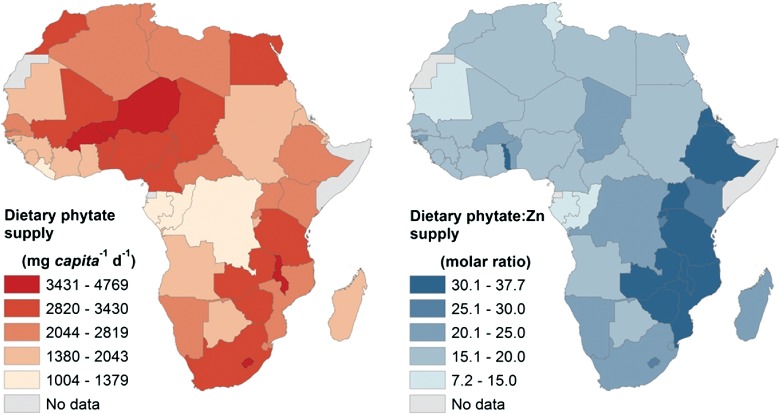
Phytate supply and phytate:Zn supply ratio for 46 African countries.

The mean supply of phytate (population-weighted) was 2770 mg capita^−1^ day^−1^, ranging from 1004 (Congo) to 4769 mg capita^−1^ day^−1^ (Niger) (Fig. 3, Tables S7 and S11). The median national supply of phytate was 2598 mg capita^−1^ day^−1^. Phytate:Zn molar ratios ranged from 7.2 (Gabon) to 37.7 (Malawi) with mean and national median ratios of 22.6 and 19.9, respectively (Table S11). The mean national contribution of ‘Cereals’ to phytate supply was 68%, with a further 17% from ‘Pulses and Beans’ and 7% from ‘Roots and Tubers’ (Table S8–S10).

### Macronutrients

The national supplies of macronutrients are given in Table S7, while the contributions of higher level food groups to macronutrient supply are given in Tables S8–S10. The mean supply of available carbohydrate (population-weighted) was 488 g capita^−1^ day^−1^, ranging from 290 (Eritrea) to 719 g capita^−1^ day^−1^ (Ghana). The mean national contribution of ‘Cereals’ to available carbohydrate supply was 55%, with ‘Roots and Tubers’ contributing a further 23%. The mean supply of protein (population-weighted) was 77 g capita^−1^ day^−1^, ranging from 30 (DRC) to 123 g capita^−1^ day^−1^ (Tunisia). The mean national contribution of ‘Cereals’ to protein supply was 50%, with ‘Animal Products’ contributing a further 25%. The mean supply of fat (population-weighted) was 58 g capita^−1^ day^−1^, ranging from 20 (Burundi) to 94 g capita^−1^ day^−1^ (Libya). The mean national contribution of ‘Animal Products’ to fat supply was 25%, with ‘Other foods’ contributing a further 33%, mainly due to cooking oils.

## Discussion

### MND risks are high in Africa

The risk of MNDs is estimated to be high in Africa based on dietary supply as calculated from FBSs and food composition tables, notably Ca (54% of the continental population), Zn (40%), Se (28%) and I (19% after accounting for iodized salt consumption). Calcium and I deficiency risks are highest in the Southern region, Zn deficiency risks are highest in the Eastern region and Se deficiency risks are highest in the Eastern and Middle regions. Many countries have high multiple MND risks. For example, in Ethiopia, the estimated deficiency risks for Ca, I, Se and Zn are 100, 64, 36 and 81%, respectively; the corresponding risks for Malawi are 61, 27, 64 and 33%, respectively (Tables1 and S6). The risk of deficiency of Fe (5%), Cu (1%) and Mg (<1%) based on supply is estimated to be lower. However, it should be noted that estimates of Fe deficiency based on supply may be less reliable than for other micronutrients because dietary Fe requirements are not normally distributed (WHO/FAO 2006, p. 156) and data for dietary requirements are highly dependent on assumptions regarding bioavailability. General methodological limitations are discussed below.

### Assumptions and caveats regarding food supply and composition data

There are general methodological caveats and assumptions to consider and emphasize when interpreting MND risks using supply data derived from FBSs and food composition data. These include the quality of the primary data and reliability of dietary requirement and intake distributions. For example, several countries have improbably high or low reported energy supplies (e.g. 3851 and 1679 kcal capita^−1^ day^−1^ in Egypt and Eritrea, respectively), probably due to inaccurate supply data. Inherent weaknesses in FBS data have been discussed extensively (FAO 2001, Hawkesworth et al. 2010a, de Haen et al. 2011, Joy et al. 2013). These include failure to account for household waste or capture data for certain foodstuffs (e.g. sweet potato has a zero supply value for Malawi in the 2009 national FBS). The approach also does not account for micronutrient or phytate losses during processing or household preparation, and is highly sensitive to food composition data (Broadley et al. 2012, Joy et al. 2013). Two specific examples (Ca and Se) are highlighted below. The reliability of dietary requirements and intake distributions is discussed subsequently.

For Ca, the FBS category ‘Freshwater Fish’ provided 40, 37 and 28% of total supply in the Eastern, Middle and Western regions, respectively, while only contributing <2% in the Southern and Northern regions, primarily due to differences in food composition values between regions. Thus, Ca concentrations for ‘Freshwater Fish’ are 2360 and 2814 mg (100 g)^−1^ FW in the Eastern and Western region composition tables, respectively, but only 86 mg (100 g)^−1^ FW in the Southern region composition table, which was also used for the Northern region. These differences in food composition data result from the inclusion or non-inclusion of small bones which have higher Ca concentrations than fish meat. Interestingly, if a ‘Freshwater Fish’ Ca concentration of 2360 mg (100 g)^−1^ FW was used in the Northern region composition table, Ca supply would increase from 599 to 796 mg capita^−1^ day^−1^, whereas a similar change for the Southern region would have minimal effect (from 353 to 357 mg capita^−1^ day^−1^) due to low supply of ‘Freshwater Fish’ in national FBSs for this region. If this higher concentration was used for all regions, the estimated risk of Ca deficiency across Africa would decrease from 54 to 48%. Conversely, if the Ca composition of ‘Freshwater Fish’ used in the Western and Eastern region composition tables was reduced to 86 mg (100 g)^−1^, Ca supply would decrease in the Eastern region from 583 to 359 mg capita^−1^ day^−1^, in the Western region from 746 to 541 mg capita^−1^ day^−1^ and in the Middle region from 764 to 492 mg capita^−1^ day^−1^. If this lower concentration was used for all regions, the estimated risk of Ca deficiency across Africa would increase markedly from 54 to 80%.

The FBS category ‘Maize’ has a Se concentration of 16 µg (100 g)^−1^ in the Western region composition table, considerably higher than in the Southern or Eastern region composition tables, which use the same value of 2.6 µg (100 g)^−1^. Chilimba et al. (2011) showed that the Se concentration of maize grain in Malawi is highly dependent on soil mineralogy, with a median concentration of 2.2 µg (100 g)^−1^, but up to 34.2 µg (100 g)^−1^ on calcareous vertisols. The effect of soil conditions on Se concentration of other food items is not known, but will clearly affect estimates of dietary Se supply greatly.

### Assumptions and caveats regarding dietary requirements and intake distributions

WHO data were used for most micronutrient requirement analyses in preference to IOM because they are based on international studies. For Ca and Zn, EAR data were chosen for diets low in animal foodstuffs but high in cereals, although Ca requirements will increase and Zn requirements decrease in sub-groups where this is not the case. For Fe, requirement data were set assuming 10% dietary bioavailability consistent with low heme Fe supply. However, considering the high estimates of phytate supply (population-weighted mean of 2770 mg capita^−1^ day^−1^) and given that acute and chronic inflammation can impair absorption of Fe from the gut, it might be more appropriate to assume 5% dietary bioavailability. At 10% bioavailability, the EAR for Fe for pregnant women is 41.2 mg capita^−1^ day^−1^ while that for non-pregnant women of childbearing age is 13.4 mg capita^−1^ day^−1^; at 5% bioavailability, the corresponding EARs are 82.4 and 26.8 mg capita^−1^ day^−1^ (WHO/FAO 2004). Assuming 5% dietary bioavailability would increase our estimated risk of dietary Fe deficiency from 5 to 43% of the total population of Africa. Iron intake requirements also increase if there are blood losses, e.g. due to helminthiasis (Stoltzfus et al. 1997), or hemolysis due to malaria (Fleming 1981). We note that a probability approach (IOM 2003, p. 73) and nationally representative food consumption surveys based on specific sex and life-stage population sub-groups might be preferable methods for assessing Fe deficiency risks, rather than national FBSs combined with EAR cut-point (WHO/FAO 2006, p. 143).

For Se, IOM data (adult EAR of 45 µg capita^−1^ day^−1^) were used in preference to WHO (adult male EAR of 28 µg capita^−1^ day^−1^) on the basis of recent research (Fairweather-Tait et al. 2011). If WHO EAR values were used, estimated risk of dietary Se deficiency across Africa would decrease from 28 to 5%.

For Western populations, the WHO adult EAR for Ca is 833 mg capita^−1^ day^−1^. Here, we adjusted for low-animal protein intake (20–40 g capita^−1^ day^−1^) to give an adult EAR of 625 mg capita^−1^ day^−1^ (WHO/FAO 2004, p. 83). However, it is possible that EARs could be adjusted further downwards in some developing countries thereby decreasing estimated risks of Ca deficiency. For example, if the mean EAR was 400 mg capita day^−1^, the estimated risk of dietary Ca deficiency would decrease from 54 to 23% of the total population of Africa; further studies on Ca requirements are required. For example, while the predominant cause of rickets worldwide is vitamin D deficiency, low-Ca intake has been found to be the primary cause in rural South African and Nigerian children (Pettifor et al. 1978, Okonofua et al. 1991, Pettifor 2004, Prentice 2013). Children with rickets in Nigeria and the Gambia responded well to Ca supplements (Thacher et al. 1999, Dibba et al. 2000). However, Jarjou et al. (2010) found that Ca supplementation of pregnant women with habitually low-Ca intake did not improve maternal bone health and their children did not have lower blood pressure at 5–10 years of age (Hawkesworth et al. 2010b). Thus, there may be some physiological adaptation to chronic low intakes of Ca (Fairweather-Tait et al. 1995, Schoenmakers et al. 2013), either through reduced urinary excretion or improved absorption efficiency.

Our study assumes that intake of each micronutrient in each country follows a normal distribution centered on mean dietary supply, with a cv of 25%. This cv was used by Wessells and Brown (2012) to estimate global Zn deficiency risk and by Joy et al. (2013) to estimate Mg deficiency risk in Africa. In reality, intake cv will vary according to population and micronutrient (Ecker and Qaim 2011). Analyses based on supply data could therefore be improved by using nationally representative individual-level dietary surveys. Increased spatial resolution of dietary data could further identify sub-national variation, such as bimodal intake distributions between rural and urban populations. Table2 shows the influence of wider (cv = 30%) and narrower (cv = 20%) distributions of intakes on estimated risks of deficiency.

**Table 2:** Estimated risks of MND across Africa at different cv, a measure of inter-individual variation of intake. CV, coefficient of variation

cv (%)	Risk of deficiency (%)						
	Calcium	Copper	Iron	Iodine	Magnesium	Selenium	Zinc
20	53	0.5	4	17	0.3	26	39
25	54	1.1	5	19	0.7	28	40
30	55	2.1	7	21	1.5	30	41

### Comparing MND risks based on supply data with other studies

Our estimates of deficiency risk are consistent with previous studies based on supply data. For example, our estimated deficiency risks of 1% for Mg and 28% for Se are similar to previous estimates of 4 (Joy et al. 2013) and 22% (Hurst et al. 2013), respectively. In this study, the risk of dietary Mg deficiency is lower due to (1) the use of 2009 rather than 2007 supply data for most countries and (2) adjustment of national EAR to take account of age and sex-specific requirements. Risk of Se deficiency is greater due to the use of USDA composition data for the FBS item ‘Coconuts – Incl copra’. Wessells and Brown (2012) estimated mean Zn and phytate supplies in sub-Saharan Africa to be 8.4 mg and 1782 g capita^−1^ day^−1^, respectively, with a phytate:Zn molar ratio of 21.3 This compares to our estimate of mean Zn and phytate supplies (population-weighted) for the Eastern, Southern, Western and Middle regions of 12.0 mg and 2728 g capita^−1^ day^−1^ and a mean phytate:Zn molar ratio of 23.8. These differences in Zn supply data are likely to originate from the food composition data. Wessells and Brown (2012) estimated that 26% of the population of sub-Saharan Africa experience inadequate Zn intake, compared to 46% in the Eastern, Southern, Western and Middle regions in our study. The primary reason for the differences in Zn deficiency risks between these two studies is that Wessells and Brown (2012) used Zn dietary requirement data from the International Zinc Nutrition Consultative Group (IZiNCG), which are based on lower requirements for most life-stage groups compared to the ‘low bioavailability’ WHO figures used here.

Approaches based on FBSs and food composition data can also be compared with household consumption data, direct intake measurements or indicators of status. For example, in Malawi, Ecker and Qaim (2011) estimated mean Fe and Zn consumption to be 19.0 and 10.2 mg capita^−1^ day^−1^, respectively, based on food consumption data from 11 280 households in the second Malawi Integrated Household Survey. In rural Malawi, Gibson and Huddle (1998) used dietary recall to estimate median Fe and Zn consumption of 14.8 and 9.0 mg capita^−1^ day^−1^, respectively, among 141 pregnant women. Through analysis of 1-day weighed diet composites prepared ‘as eaten’, Siyame et al. (2014) estimated Fe and Zn consumption to be 21.0 and 5.7 mg capita^−1^ day^−1^, respectively, and high phytate:Zn molar ratios (median 20.0) in the diets of 113 adult women. These values are generally lower than our estimates for Fe and Zn supplies in Malawi (29.1 and 11.8 mg capita^−1^ day^−1^, respectively) and phytate:Zn molar ratio (37.7); this was the highest ratio observed for Africa based on supply data and was driven by the high phytate supply (4510 mg capita^−1^ day^−1^), approximately 65% of which comes from maize. In general, it is expected that FBSs will overestimate supply, including that of micronutrients and phytate, compared to dietary surveys or analyzed diet composites given that losses during food processing and preparation and household waste are not considered. Andersson et al. (2012) estimated that 40% of the population of Africa are I deficient, defined as urinary iodine concentration <100 µg L^−1^. This is greater than our estimate of 19% I deficiency based on supply, which could be due to overestimates of I supply from FBS items or salt.

### The role of food-based strategies to alleviate MNDs

A key question facing agriculture and, in particular, plant nutrition is its role in increasing the dietary supply of bioavailable micronutrients within the wider context of dietary diversification or direct fortification of foods. Here, we sought to determine in theoretical terms the effect of dietary diversification on micronutrient supplies by analyzing changes to food supply data. Such an approach can be used at multiple scales and we illustrate its use at a national scale for Malawi, Nigeria and South Africa.

For each country, the 92 FBS items were assigned to a taxonomy of 17 ‘higher level’ food groups (defined in Table S8). The micronutrient and macronutrient composition of these food groups was calculated at a national scale, weighted according to the supply of the constituent items within each group and the appropriate regional food composition table. In addition to national-level EARs, national mean RNIs were calculated using WHO intake requirement data for Ca, Fe, I, Mg and Zn (WHO/FAO 2004) and IOM data for Cu and Se (IOM 2000, 2002) (Table S5). Lower RNIs (LRNIs) were calculated as EAR-(RNI-EAR). The LRNI defines an intake of a specific micronutrient that is sufficient only for approximately 2% of an age and sex-defined population sub-group; intakes below the LRNI carry a very high risk of deficiency. Tolerable upper levels (TULs) of intake for Ca and Zn were taken from WHO/FAO (2004), for Fe and Cu from IOM (2000) and for Se from IOM (2002) (Tables S4 and S5). The TUL is the maximum intake of a specific micronutrient from food, water and supplements that is unlikely to induce adverse health effects in approximately 97% of apparently healthy individuals (WHO/FAO 2004). Although a TUL for I has not been published by WHO, the IOM TUL of 1100 µg capita^−1^ day^−1^ was not adopted here due to the greater risk of hyperthyroidism in countries with long-standing I deficiency, for example, as in Zimbabwe in the early 1990s (Todd et al. 1995). A TUL for I of 600 µg capita^−1^ day^−1^ was therefore drawn from The European Commission Scientific Committee on Food (EU SCF 2002). A TUL for Mg has not been set by the WHO or IOM as there is no evidence of harm from excessively high intake from food. A TUL for Ca for children and adolescents and for Zn for children has not been set and were assumed to be the same as the other age categories. The proportion of the population of each country falling into the following categories was determined: ‘below LRNI’, ‘between LRNI and EAR’, ‘between EAR and RNI’, ‘between RNI and TUL’ and ‘above TUL’. In order to represent potential health consequences of different intake levels, categories were assigned an arbitrary error weighting as follows: ‘above TUL’ = 1, ‘below LRNI’ = 0.975, ‘between LRNI and EAR’ = 0.75 and ‘between EAR and RNI’ = 0.25. The category ‘between RNI and TUL’ was assumed to have no error, thereby indicating sufficient supply. Weightings were applied equally to all micronutrients and countries. For each micronutrient, the errors arising from each category were summed to give a ‘Micronutrient Error’ score between 0 and 1. A cumulative ‘Total Error’ was calculated as the sum of all ‘Micronutrient Error’ scores.

The supply of each of the 17 higher level food groups was allowed to increase or decrease by one- to two-fold, relative to 2009 supply. The Solver function of Microsoft Excel 2010 (Microsoft Corporation) was used to minimize the ‘Total Error’ term. Energy supply was constrained to remain below the starting value and above a level defined as national mean daily energy requirement, which is the quantity of food energy needed to balance energy expenditure and maintain body size, body composition and a level of necessary and desirable physical activity consistent with long-term good health, assuming light work (Table S5) (FAO/WHO 2001). A standard physical activity level of 1.9 and mean body weights of 70 and 65 kg were assumed for adult men and women, respectively; WHO reference weights were used for children and adolescents (FAO/WHO 2001). No upper constraint was placed on supplies of carbohydrate, protein and fiber, but carbohydrate and fiber were prevented from decreasing below the national mean recommended dietary allowance (RDA) (equivalent to RNI; IOM 2005), and protein from below a safe level of intake, defined as sufficient for approximately 97% of the population (WHO/FAO 2002a). Pregnant and lactating women were assumed to require an additional 14 g capita^−1^ day^−1^ of protein. Fiber requirements were based on the adequate intakes, and carbohydrate requirements on RDAs (IOM 2005). Fat intake recommendations were derived from acceptable macronutrient distribution ranges (AMDRs; IOM 2005, p. 941). The AMDR is the recommended proportion of energy intake, based on a healthy diet and adequate exercise, with the aim of minimizing risk of chronic disease over the long-term while allowing adequate consumption levels of essential nutrients. The AMDR was set at 25% for adults, 30% for adolescents and 35% for children (IOM 2005, p. 941). Recommended fat intake was then calculated from recommended energy intakes, assuming that the energy density of fat was 9 kcal g^−1^. Where initial fat supply exceeded national mean recommended intakes (e.g. in South Africa, the initial 2009 fat supply of 85 g capita^−1^ day^−1^ exceeded the mean recommended intake of 77 g capita^−1^ day^−1^), fat supply was constrained so as not to increase further. Salt (NaCl) supply was not allowed to increase beyond 5 g capita^−1^ day^−1^, the intake target set by WHO (WHO/FAO 2002b, p 90). Nigeria (10.3 g capita^−1^ day^−1^) and Malawi (8.2 g capita^−1^ day^−1^) both had high initial supplies of salt and were therefore constrained not to increase further.

**Figure 4:**
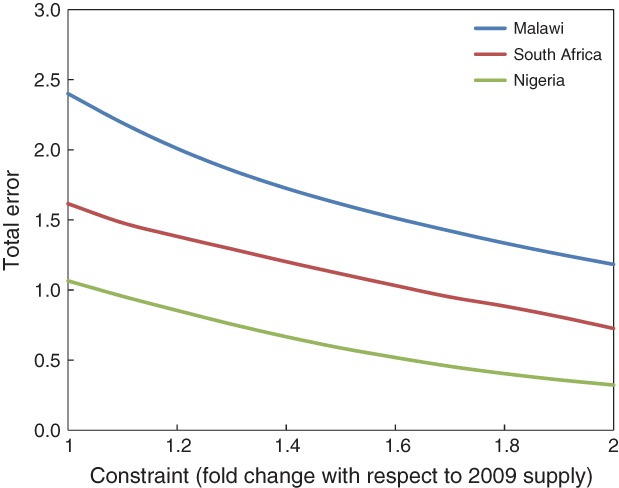
‘Total Error’ output from the Solver model used to optimize dietary diversification for Malawi, Nigeria and South Africa.

For Malawi, the ‘Total Error’ term was 2.40 for 2009 supply data. The main sources of error were an undersupply of Ca (0.59), Se (0.61), Zn (0.39), fat (0.43) and I (0.35). Using a constraint of 1.3-fold, the ‘Total Error’ term decreased to 1.86, indicating that the average Malawi diet could theoretically be ‘improved’ in terms of micronutrient supply through dietary diversification (Fig. 4). This improvement occurred because the supply of several food groups increased to their constrained maxima (i.e. 130% of 2009 supply), including animal-sourced foods (‘Dairy’, ‘Eggs’, ‘Fish’ and ‘Meat’), ‘Pulses and Beans’ and ‘Vegetables’. ‘Maize’ and ‘Cassava’, the two largest supply groups by weight, decreased by 4 and 23%, respectively. Using a constraint of 1.6-fold, the ‘Total Error’ term decreased further to 1.51, with Ca, Se, Zn, I and fat all still contributing errors, and with the same trends in the supply of the different food groups. A constraint of twofold decreased the ‘Total Error’ term to 1.18, with Zn as the main source of error (0.30), followed by I (0.28), Se (0.27) and fat (0.26). Therefore, although supply of food groups varied by 100%, macronutrient and micronutrient supply needs were still not fully met and it is not immediately apparent how Zn supply could be increased relative to Ca and Se without increasing total energy supply.

For Nigeria, the ‘Total Error’ term was 1.07 for 2009 supply data. The main sources of error were Fe (0.64), Ca (0.28) and Zn (0.12). The errors for Ca and Zn resulted from insufficient dietary supply, with 27 and 10% respectively, being below the EAR. The error for Fe, however, resulted from a high proportion of the population (63%) being above the TUL due to a supply of 47 mg capita^−1^ day^−1^ compared to the mean population TUL of 43 mg capita^−1^ day^−1^. Using a constraint of 1.3-fold, the ‘Total Error’ term decreased to 0.76, mainly through a decreased supply of Fe-rich food groups including millet and sorghum, but also through increased supply of Ca-rich groups including dairy and fish. Using constraints of 1.6- and 2-fold, the ‘Total Error’ term decreased to 0.53 and 0.32, respectively.

Dietary excess of Fe has previously been reported for black populations in South Africa and Zimbabwe due to high consumption of traditional beers which are rich in bioavailable Fe due to fermentation and cooking in iron pots (Bothwell et al. 1964). Indeed, sorghum and millet, which can both be used to produce traditional beers, provide a relatively high supply in Nigeria (101 and 95 g capita day^−1^, respectively) which, combined with their relatively high Fe concentrations, supply 8.8 mg capita^−1^ day^−1^ and 13.6 mg capita^−1^ day^−1^ of Fe. However, as estimated energy supply in Nigeria was improbably high (3373 kcal capita^−1^ day^−1^), the supply data are likely to have been overestimated. Contaminant Fe from soil is a potentially large source of dietary intake *via* a wide range of crops (Harvey et al. 2000). Typical soil Fe concentrations in Malawi ranged from 25 000 to 45 000 mg kg^−1^ as compared to Fe concentrations in (uncontaminated) maize flour of approximately 30 mg kg^−1^ (Chilimba et al., unpublished observations). Thus, 0.1% soil contamination would approximately double Fe intakes from maize, primarily in ferric (Fe^3+^) oxide forms whose bioavailability is uncertain. Siyame et al. (2014) found low a prevalence of Fe deficiency among Malawian women despite a cereal-based diet and a high prevalence of Zn deficiency. Based on the data from analyzed diet composites, they suggested that contaminant Fe may have joined the common non-heme Fe pool and become available for absorption.

For South Africa, the ‘Total Error’ term was 1.62 for 2009 supply data. The main source of error was an undersupply of Ca (0.97), with smaller undersupplies of I (0.29), Se (0.16), Zn (0.10) and Fe (0.09). Using a constraint of 1.3-fold, the ‘Total Error’ term decreased to 1.29 but undersupply of Ca (0.91) still dominated. The supply of Ca-rich food groups, including animal-sourced foods (‘Dairy’, ‘Eggs’, ‘Fish’ and ‘Meat’) and ‘Pulses and Beans’, all increased to their constrained maxima. Using a constraint of twofold, the ‘Total Error’ term decreased to 0.73 and the undersupply of Ca (0.54) still dominated, with 25% of the population below the national mean LRNI for this element, and 31% between the LRNI and EAR.

Clearly, the potential for dietary diversification to meet all micronutrient supply requirements is challenging in the absence of wholesale dietary changes and direct food fortification strategies may need to be considered. In dietary surveys of UK populations, 8% of females and 4% of males aged 19–64 years consume less than the LRNI of 400 mg Ca capita^−1^ day^−1^, rising to 18 and 7% of females and males aged 11–18 years, respectively (Department of Health/Food Standards Agency 2011), despite a large consumption of dairy products which would be unachievable in the short term for many African households. In the UK, almost three-quarters of Ca intake comes from dairy products and cereals (Broadley and White 2010); the former account for 46% of Ca intake for females and 41% for males. Cereal sources account for 28% of Ca intake for females and 32% for males even though cereal grains are generally low in Ca. This high contribution of cereals to dietary Ca intake in the United Kingdom is partly due to legislation requiring processed wheat flour to be fortified with Ca and Fe and the ‘B vitamins’, thiamine and nicotinic acid. Processed flour must therefore contain Ca within the range 235–390 mg (100 g)^−1^ flour, with the exceptions of wholemeal flour and self-raising flour (>200 mg Ca (100 g)^−1^) and wheat malt flour. Although flour fortification programs exist or are under development in several African countries, levels of compliance are often poor (Yusufali et al. 2012) and none exist for Ca. Fortification at the processing stage will not benefit subsistence households who mill grain at home or at small community mills which, in countries such as Malawi, comprise the majority of households (Dorward and Chirwa 2011).

### The role of agricultural strategies to alleviate MNDs

This discussion focuses primarily on Ca, Zn and Se due to their greater risks of deficiency. By contrast, the risks of Cu and Mg deficiency appear to be lower at a continental scale; Mg deficiency risks and the role of plant nutrition are discussed elsewhere (Broadley and White 2010, Joy et al. 2013). For Fe, approaches to produce crops with increased bioavailable Fe concentration through breeding or agronomy have been widely reviewed (White and Broadley 2009, Zhao and Shewry 2011, Murgia et al. 2012). The main policy to address dietary I deficiency is the use of iodized salt (Mason et al. 2005, Pearce et al. 2013) and agriculture is likely to have a lesser general role. However, to protect against chronic diseases including high blood pressure, the WHO has set a target population average salt intake of 5 g day^−1^ (WHO 2007), which could create conflict with the public health goal of meeting dietary I requirements. If all countries in Africa had average per capita salt intake of 5 g day^−1^, mean (population-weighted) per capita supply of dietary I would fall from 171 to 125 µg day^−1^, with the estimated risk of dietary I deficiency increasing from 19 to 35% of the population. In addition, increased Na intake may increase urinary excretion of Ca, with a 1 g increase in Na intake increasing Ca excretion by approximately 30 mg (Shortt et al. 1988). Agronomic approaches based on fertilization/irrigation (Cao et al. 1994, Ren et al. 2008), breeding (White and Broadley 2009) or livestock salt licks (Phillips 1997) could potentially be of use in increasing I supply in the food chain, although published information is sparse.

Agronomic biofortification can play a major role in alleviating dietary Se and Zn deficiencies. Increasing Se concentrations in cereal grain by incorporating Se into granular nitrogen fertilizer in its selenate form provides a simple, cost-effective fertilizer-based solution which can be applied in many contexts (Broadley et al. 2006, 2010), including Malawi (Chilimba et al. 2012a, 2012b). In 1984, Finland adopted Se fertilization as a national public health measure, leading to increases in dietary intake of Se, a successful initiative which has continued for almost 30 years (Alfthan et al. 2011). For Zn, fertilization of cereals via foliar sprays or addition to the soil is a possible strategy (White and Broadley 2011) which has proved successful in mitigating Zn deficiencies in Turkey and India and has shown potential in other countries (Cakmak 2004, 2009, Zou et al. 2012). In cereals, grain Zn concentration tends to be correlated with grain protein concentration (Zhao et al. 2009, Gomez-Becerra et al. 2010), with grain Zn concentration increasing at higher N-fertilizer application rates, with application of cattle manure and other organic amendments, through rotation with legumes and by using late foliar applications of soluble Zn such as ZnSO_4_ (Kutman et al. 2011, Manzeke et al. 2012, Xue et al. 2012, Zou et al. 2012). Fertilization is unlikely to be an effective biofortification strategy for Ca in cereal crops due to plant and soil factors (Broadley and White 2010). As Ca is highly immobile in the phloem, its translocation to the grain is limited. However, common Ca-based fertilizers including lime (CaO and CaCO_3_), gypsum (CaSO_4_), Ca-phosphates, Ca(NO_3_)_2_ and a range of foliar products could potentially be used to increase Ca concentration in crops such as leafy vegetables, while addition of manure and charcoal can increase soil Ca concentration and decrease leaching in highly weathered tropical soils (Lehmann et al. 2003, Steiner et al. 2007). However, a reliable agronomic Ca biofortification strategy has not yet been developed.

Plant breeding offers another feasible biofortification strategy to increase the dietary supply of micronutrients essential in the human diet by (1) increasing micronutrient uptake by crops from the soil, (2) increasing the their translocation from leaves to the grain or fruit of crops or (3) reducing the biosynthesis of compounds which inhibit absorption of micronutrients in the gut, such as phytate (White and Broadley 2009, Bouis et al. 2011). For example, increasing the uptake and translocation of Fe and Zn through conventional breeding is a priority of the HarvestPlus program, which has set target Fe and Zn concentrations of 15 and 28 µg g^−1^ dry matter (DM) in polished rice, 59 and 38 µg g^−1^ DM in wheat, 60 and 38 µg g^−1^ DM in maize, 88 and 66 µg g^−1^ DM in pearl millet, 107 and 56 µg g^−1^ DM in beans, 45 and 34 µg g^−1^ DM in cassava roots and 85 and 70 µg g^−1^ DM in the roots of sweet potato, respectively (Bouis and Welch 2010, White and Broadley 2011). If these breeding targets were met for maize and beans and uptake of improved crop varieties was universal, mean (population-weighted) per capita supply of dietary Zn would increase from 12.9 to 15.3 mg day^−1^ (Table S12) and mean estimated risk of Zn deficiency in Africa would decrease from 40 to 23%. Mean phytate:Zn molar ratio would also decrease from 22.6 to 18.3 and would be >15 in 38 of the 46 countries instead of the current 42. If HarvestPlus breeding targets for Zn were met for rice, wheat, maize, pearl millet, beans, cassava roots (adjusted for 60% moisture, USDA 2011) and sweet potato (adjusted for 77% moisture, USDA 2011), mean (population-weighted) per capita supply of dietary Zn would increase to 22.7 mg day^−1^ and the estimated risk of deficiency in Africa would decrease to just 4%. Only seven countries (Eritrea, Ethiopia, Lesotho, Malawi, Togo, Zambia and Zimbabwe) would have a mean phytate:Zn molar ratio of >15.

For Ca, there might be a potential for genetic biofortification based on the observed genotypic variation in its concentration in the leaves of *Brassica* spp., onion, spinach and chickpea, the roots of carrot and cassava and the fruits of plantain and plum (reviewed by Broadley and White 2010). Transgenic strategies might also be possible based on the overexpression of vacuolar-localized Ca^2+^/H^+^-antiporters (e.g. CAX1) and Ca^2+^-binding proteins such as calreticulin. Interestingly, if Ca concentrations of the single FBS category ‘Vegetables, other’ could be doubled in all regions through breeding or agronomy, the mean (population-weighted) supply of dietary Ca would increase from 645 to 683 mg capita^−1^ day^−1^ and the mean estimated risk of Ca deficiency would decrease from 54 to 48%. However, it should be noted that the FBS category ‘Vegetables, other’ encompasses a wide range of leafy and non-leafy vegetables which vary in Ca concentration and bioavailability, e.g. due to varying levels of oxalic acid. It is currently thought that breeding crops for increased Se uptake using conventional means is unlikely to be effective based on observed genetic variation among cereal germplasm collections (Lyons et al. 2005, Zhao et al. 2009). However, transgenic breeding strategies might be possible in the longer term based on wider variation in Se accumulation patterns among flowering plants (Broadley et al. 2006).

In terms of reducing the biosynthesis of compounds which inhibit absorption of micronutrients in the gut, the compounds which have received the most attention to date are the phytic acid salts, referred to collectively as phytate. Phytate is a potent inhibitor of Fe, Mg and Zn absorption in the human intestine (Bohn et al. 2004, Miller et al. 2007, Hurrell and Egli 2010). Given the very high levels of phytate intake estimated from FBS and food phytate composition data in our study (population-weighted mean of 2770 mg capita^−1^ day^−1^, range 1004–4769) and a mean phytate:Zn molar ratio of 22.6, it may be appropriate to consider lower phytate cereals and beans to complement other agricultural strategies such as biofortification through micronutrient fertilization or lower phytate breeding (White and Broadley 2009, Bouis and Welch 2010).

From our analysis of higher level food groups, 68% of total dietary phytate supply in Africa is from ‘Cereals’, with 17% from ‘Pulses and Beans’. Many of these crops (e.g. maize, wheat, rice and common bean) have potential for low phytate breeding based on extensive genetic variation in seed phytate concentration among germplasm collections (reviewed by White and Broadley 2009). For example, greater than threefold variation in grain phytate was observed among 60 maize (Mladenović Drinić et al. 2009) and 28 wheat genotypes (Welch et al. 2005), while greater than twofold variation in seed phytate was observed among 18 genotypes of common bean (House et al. 2002). Crosses derived from lines with contrasting phytate levels have been used to identify single and/or quantitative genetic loci for use in breeding programs. Natural and induced mutants with very low phytate concentrations (*low phytic acid*, *lpa*, mutants) have also been reported in a range of crops including wheat, maize, rice and soybean (reviewed by Raboy 2007, Bohn et al. 2008). While reduced seed phytate may potentially reduce the agronomic performance of crops, it has been suggested that deleterious pleotropic effects of the *lpa* mutation can be overcome by breeding in wheat (Guttieri et al. 2006). Furthermore, recent studies on *lpa* mutants of common bean found no decrease in agronomic performance in the field (Campion et al. 2013). Transgenic strategies to reduce phytate concentrations in crops include reducing the expression of genes encoding enzymes involved in the biosynthesis or sequestration of IP_6_, as demonstrated in several crops species, including rice (Kuwano et al. 2006) and maize (Shi et al. 2007). It is also possible to overexpress phytase enzymes to release phosphate groups from phytate using microorganism-derived (Chen et al. 2008 in maize) and plant-derived (Holme et al. 2012 in barley) phytases.

If the phytate concentrations of maize, wheat, rice, sorghum, millet and beans were halved compared to existing levels and universal uptake was achieved, the mean (population-weighted) supply of dietary phytate would decrease from 2770 to 1782 mg capita^−1^ day^−1^. Mean (population-weighted) phytate:Zn molar ratio would be 14.6 and would be >15 in only 16 of the 46 countries studied. However, in addition to the possible reduced agronomic performance of crops, any attempts to reduce phytate in human diets should proceed with caution because dietary phytate intake has been linked to health benefits including reduced risk of cancer and osteoporosis (Vucenik and Shamsuddin 2006, López-González et al. 2008, Kumar et al. 2010). While this study highlights the importance of phytate as a contributory factor to dietary mineral deficiencies, it may also be informative to consider other nutrient–nutrient interactions such as vitamin D–Ca (Jones et al. 1998), ascorbic acid–Fe (Hallberg et al. 1989) or Fe–Ca (Prentice 2013).

## Conclusion

MNDs are a widespread public health problem affecting billions of people worldwide. While data and methodologies exist to determine the prevalence of some MNDs, there is a dearth of estimates of country-level prevalence rates for others. We combined data from FAO FBSs, regional food composition tables and salt iodization to estimate dietary mineral supplies of Ca, Cu, Fe, I, Mg, Se and Zn for 46 countries in Africa. We then estimated the risk of MNDs due to inadequate dietary intake using an EAR ‘cut-point’ approach. There are high risks of MNDs in Africa for Ca (54% of population), Zn (40%), Se (28%) and I (19%), whereas the risks of deficiency for Fe (5%), Cu (1%) and Mg (<1%) are much lower.

Of course, these estimated MND risks are contingent on major caveats and assumptions, including the quality of the input data and the validity of the dietary requirements used. For example, the estimated Fe deficiency risk is based on intake requirements which assume that 10% of Fe is bioavailable in food. Estimates of Fe deficiency risks are much greater (43%) if Fe absorption is assumed to be 5%, which is plausible based on high phytate supply and other factors. This example illustrates that, while supply-based approaches are a useful framework, they must be interpreted with caution. Further studies are needed to determine how, and to what extent, our methodology can be used, especially for Fe deficiency.

We have shown how a supply-based methodology can provide evidence to address crucial questions regarding how to address hidden hunger in Africa or elsewhere. To demonstrate this, we assessed the possible influence of dietary diversification and dietary change within realistic boundaries by simulating an alteration of the food supply in Malawi, Nigeria and South Africa. This analysis showed – unsurprisingly – that if the current consumption of cereals and cassava was partially replaced by other food groups, especially animal products and vegetables, the risk of dietary deficiencies could be reduced. However, dietary diversification on its own is still unlikely to meet all mineral requirements in the region.

Given the growing interest in using agriculture-based strategies to address MNDs, we also simulated a scenario in which current rice, wheat, maize, pearl millet, beans, cassava and sweet potato varieties are biofortified with Zn. As breeding of such varieties is currently taking place, knowing whether this is a potentially effective intervention is important. Our analysis suggests that such an improvement in the dietary quality of key crops could reduce the risk of inadequate dietary Zn supply in Africa from 40% to just 4%. A complementary intervention is the application of mineral fertilizer to staple food crops to increase the supply and uptake of crucial nutrients. Further studies of such approaches are needed, in particular regarding the potential coverage and costs of interventions, whether through breeding or fertilizer application. Finally, we believe that Tables S1–S12 provide a useful ‘audit trail’ so that: (1) potential errors can be identified, (2) additional nutrients (or antinutrients/contaminants) can be added to the analysis and (3) alternative food composition data can be incorporated at different spatial scales.

## Abbreviations

AMDR: acceptable macronutrient distribution range
CBR: crude birth rate
cv: coefficient of variation
DRC: Democratic Republic of the Congo
DW: dry-weight
EAR: estimated average requirement
FAO: Food and Agriculture Organization
FBS: food balance sheet
FW: fresh-weight
IOM: Institute of Medicine
LRNI: lower reference nutrient intake
MND: micronutrient deficiency
RDA: recommended dietary allowance
RNI: reference nutrient intake
TUL: tolerable upper level
UN: United Nations
WHO: World Health Organization

## Supporting Information

Table S1 FAO names and subregions of the 46 African countries studied (http://unstats.un.org/unsd/methods/m49/m49regin.htm)

Table S2 Food mineral composition data from literature sources, used in conjunction with FBSs to estimate dietary mineral availability.

Table S3 National salt I concentration and regional salt consumption estimates for 46 African countries. Salt concentration data from UNICEF () (www.childinfo.org), consumption data from ICCIDD () (http://www.iodinenetworknet/Resources_Region.htm).

Table S4 Nutrient requirements per capita.

Table S5 National mean (population-weighted) macronutrient and micronutrient requirement data for 46 African countries.

Table S6 National mean EAR, *per capita* micronutrient supply (standard deviation, sd, calculated assuming 25% inter-individual variation in intake) and estimated risk of deficiency for 46 African countries.

Table S7 Energy, macronutrient and micronutrient supply data calculated from food composition tables and FAO FBS data (FAO 2012b) (http://faostat.fao.org).

Table S8 Higher level food groupings used to study contribution of different components of the diet to national mineral supply.

Table S9 Total nutrient supply by higher level food group for 46 African countries and aggregated by UN sub-region.

Table S10 Percentage of total nutrient supply by higher level food group for 46 African countries and aggregated by UN sub-region.

Table S11 Phytate supply, phytate:Zn, phytate:Fe, phytate:Ca and phytate:Mg data calculated from food composition tables and FAO FBSs (FAO 2012b) (http://faostat.fao.org).

Table S12 Zinc, Ca and phytate supply data, and phyate:Zn and phytate:Ca molar ratios under biofortification and low-phytate breeding scenarios.
